# A Rare Case of Neuroendocrine Prostate Cancer Detected on 68Ga – DOTANOC Positron Emission Tomography/Computed Tomography (PET/CT)

**DOI:** 10.7759/cureus.52375

**Published:** 2024-01-16

**Authors:** Abhishek Kumar, Aaditya Prakash, Amitabh Kumar Upadhyay, Bhola Kumar, Sujata Mitra

**Affiliations:** 1 Nuclear Medicine, Tata Main Hospital, Jamshedpur, IND; 2 Radiation Oncology, Tata Main Hospital, Jamshedpur, IND; 3 Medical Oncology, Tata Main Hospital, Jamshedpur, IND

**Keywords:** neuroendocrine tumor, pet/ct, positron emission tomography, prostate cancer, nepc, 68ga - dotanoc

## Abstract

Prostate cancer is one of the most common malignancies affecting elderly men worldwide and the fifth leading cause of cancer death in men. Prostate cancer includes many histological variants with the prostatic acinar adenocarcinoma variant accounting for the majority of the diagnosed cases. Other less common histological variants are broadly classified as non-acinar carcinomas. One of the non-acinar carcinoma variants is neuroendocrine prostate cancer (NEPC). NEPC can emerge as a mechanism of treatment resistance in castration-resistant conventional prostate cancer and can also rarely be seen as a primary histological form at the time of initial diagnosis. Like other non-acinar carcinoma variants of prostate cancer, NEPC is also an aggressive variant with associated poor prognosis. Neuroendocrine tumors (NETs) are characterized by the expression of somatostatin receptors (SSTRs). Positron emission tomography/computed tomography (PET/CT) using radiolabeled somatostatin analogs like DOTANOC have been used to detect and stage these NETs. These radiolabeled somatostatin analogs also provide the option of treatment of these tumors and have been used in peptide receptor radionuclide therapy of these tumors. NEPC being a neuroendocrine malignancy also expresses SSTRs and hence can be detected with PET/CT radiotracers like 68Gallium-labeled somatostatin analogs. We here report a case of metastatic treatment-emergent NEPC detected on 68Ga - DOTANOC PET/CT.

## Introduction

Prostate cancer is the second most common malignancy and the fifth leading cause of cancer death among men worldwide. It is a malignancy primarily afflicting elderly men. However, it can also occur in men below 65 years of age. Other risk factors associated with prostate cancer are family history and black race [1]. The majority of men diagnosed with prostate cancer are asymptomatic at the time of diagnosis. However, prostate cancer may present with nonspecific lower urinary tract symptoms like hematuria. Other symptoms like frequency, urgency, nocturia, and hesitancy can also be seen; however, these are more commonly seen in benign conditions such as benign prostatic hyperplasia. Bone pain can also be a presenting complaint in case of patients diagnosed with metastatic skeletal disease at onset. The serum prostate-specific antigen (PSA) level is a commonly used initial test in the diagnosis of prostate cancer. The possibility of prostate cancer increases with higher values of PSA levels. However, there is no single threshold value of PSA to decide the diagnosis of prostate cancer.

Moreover, raised PSA levels can also be seen in benign conditions like prostatitis. Digital rectal examination (DRE) is used to detect enlarged prostate, asymmetry, and prostate nodules, which guides further evaluation. Suspicious DRE findings, combined with PSA levels, help guide the clinician in deciding whether there is a need for a biopsy [2]. However, prostate cancer is not always detected on DRE and, hence, not a recommended standalone screening modality for prostate cancer [3]. Imaging modalities like transrectal ultrasonography (TRUS) and magnetic resonance imaging (MRI) have been used to improve the accuracy of prostate biopsies [4]. Positron emission tomography (PET) in conjunction with computed tomography (CT) and MRI are also being used now for imaging-guided biopsy of prostate lesions [5]. On biopsy, acinar adenocarcinoma is the most common variant of prostate cancer, accounting for more than 90% of the cases. Other non-acinar carcinoma histological variants account for 5-10% of cases of prostate cancer [6]. These histological variants include neuroendocrine prostate cancer, sarcomatoid carcinoma, ductal adenocarcinoma, urothelial carcinoma, squamous and adenosquamous carcinoma, and basal cell carcinoma. After confirmation of prostate cancer on biopsy, imaging is undertaken for staging of the disease and deciding treatment modalities available for the patient. MRI is the recommended modality for locoregional evaluation and guiding biopsy in cases of prostate cancer [7].

CT and whole-body bone scans have been used for staging prostate cancer. However, now, prostate-specific membrane antigen (PSMA) PET/CT is being increasingly used in staging and response evaluation of adenocarcinoma prostate [8]. Another PET radiotracer 18F - fluorodeoxyglucose (FDG) has also been used in the staging of other aggressive non-acinar histologic variants of prostate cancer. NEPC is an aggressive variant of prostate cancer and 18F - FDG and 68Ga - DOTANOC PET/CT have been used in the staging of these tumors. 18F - FDG is used in cases of high-grade neuroendocrine tumors and 68Ga - DOTANOC in cases of small-cell or large-cell NEPC and well-differentiated neuroendocrine tumors of the prostate that express SSTRs. Depending on the Gleason score, stage, and condition of the patient in cases of adenocarcinoma prostate, surgical treatment options include radical prostatectomy, robotic or laparoscopic prostatectomy, and transurethral resection of the prostate (TURP). Radiotherapy (RT) is also used in the treatment of prostate cancer either in the form of external beam RT including stereotactic RT and brachytherapy. Adenocarcinoma variants of prostate cancer are androgen-dependent, and hormonal therapy treatment options like bilateral orchidectomy and androgen deprivation therapy (ADT) drugs are also used. In metastatic stage IV cases of prostate cancer, chemotherapy is also used, the most widely used chemotherapy agent being docetaxel. When all treatment options are exhausted, then peptide receptor radionuclide therapy (PRRT) is an option for treatment in these patients. In PRRT, the patient receives an intravenous injection of radiopharmaceutical which binds to receptors on the tumor cells and kills the tumor cells with radiation.

## Case presentation

**Figure 1:**
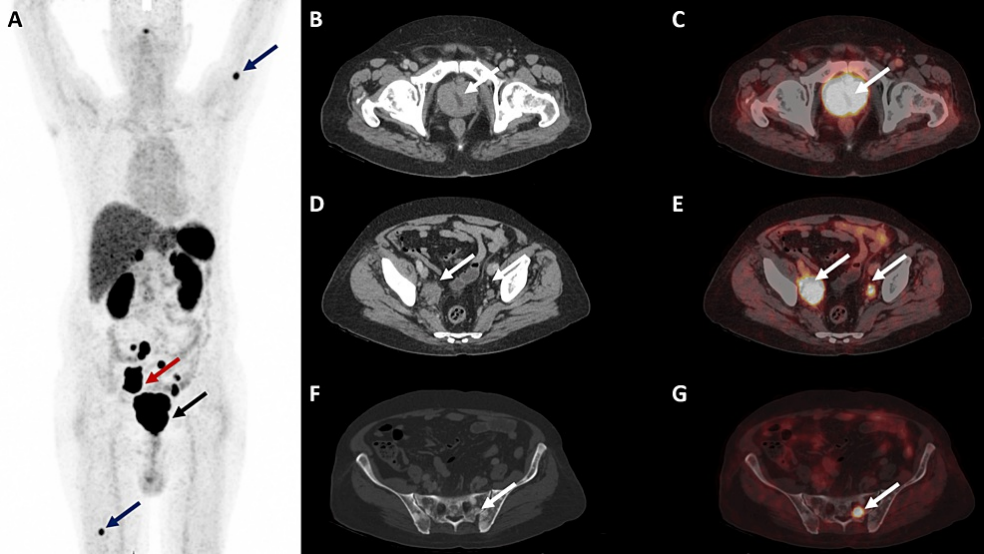
68Ga - DOTANOC PET/CT images of the patient (A) MIP; (B, D, & F) axial view of CT; (C, E, & G) axial view of 68Ga - DOTANOC PET/CT fusion. Image A shows DOTANOC uptake in the region of the prostate (black arrow), pelvic lymph nodes (red arrow), and skeletal metastatic sites in the region of the left humerus and right femur (blue arrows). Images B & C reveal prostate mass with DOTANOC uptake suggesting somatostatin receptor-expressing neoplastic disease (white arrows). Images D & E show bilateral iliac lymph nodes with DOTANOC uptake (white arrows). Images F & G show a sclerotic lesion involving the sacrum with DOTANOC uptake (white arrows). MIP: Maximal intensity projection; 68Ga-DOTANOC PET/CT: 68Gallium-labeled DOTANOC positron emission tomography/computed tomography.

We report a case of biopsy-proven NEPC with nodal and skeletal metastases. The index case is a 65-year-old man who initially presented with complaints of hematuria. At initial presentation, serum PSA was raised (7.8 ng/mL), and DRE revealed an enlarged, hard, nodular prostate gland. TRUS-guided biopsy of the prostate confirmed the diagnosis of adenocarcinoma carcinoma of the prostate with a Gleason score of 8 (4 + 4). CT thorax and abdomen with whole body bone scan were negative for nodal and distant metastases. The patient did not want to undergo surgical treatment after initial staging and workup. He received a definitive dose of radiation therapy at the local site. The patient was put on ADT and was followed up with serum PSA levels. Approximately 18 months after RT, the patient came to the outpatient unit with a complaint of bone pain. The serum PSA level was raised (168.2 ng/mL), and a whole-body bone scan revealed multiple skeletal metastases. CT thorax and abdomen confirmed skeletal metastasis in the form of sclerotic lesions involving multiple skeletal sites. Diagnosis of metastatic castration-resistant prostate cancer (mCRPC) was established in conjunction with biochemical tests, and the patient was started on docetaxel-based palliative chemotherapy at 75 mg/m^2^, a total of six cycles at three weeks intervals. The patient responded well, symptoms of pain were alleviated, and the PSA level came back within the normal range. The patient was continued on ADT and kept on follow-up with serum PSA level monitoring. Again, after a period of approximately 12 months, the patient came back with complaints of hematuria and pelvic bone pain. DRE revealed a hard, nodular prostate; however, the PSA level was within the normal range (2.8 ng/mL). TRUS-guided biopsy was undertaken. Histopathology, in conjunction with IHC, confirmed the diagnosis of small-cell NEPC. 68Ga - DOTANOC PET/CT was conducted for staging of the disease after obtaining written consent from the patient. The PET/CT study was analyzed, and maximum standardized uptake values (SUVmax) were obtained. Findings (Figures 1A-1G) revealed DOTANOC avid soft tissue lesions involving the entire prostate gland (SUVmax 67.4). Pelvic lymph nodes (SUVmax 50.1) and skeletal sclerotic lesions (SUVmax 27.5) were also noted with increased DOTANOC uptake. A background SUVmax value of 3.1 was noted. These findings established the diagnosis of stage IV NEPC. Subsequently, the patient received a cisplatin-based palliative chemotherapy regimen. However, after three months, the patient opted out of therapy due to further deterioration of his condition.

## Discussion

Prostate cancer is one of the most common malignancies and the fifth leading cause of death in men worldwide. Adenocarcinoma, the most typical variant of prostate cancer, is typically androgen-dependent; however, it can exhibit androgen resistance. Surgical castration and ADT like GnRH agonists and hormonal therapy with abiraterone have been used in the management of prostate cancer. However, despite these treatment options, prostate cancer progresses and metastasizes within two to three years. These cases of prostate cancer demonstrating androgen resistance as per serum PSA and testosterone levels are termed castration-resistant prostate cancer (CRPC). The diagnosis of CRPC is not dependent only on the progression of symptoms; it is defined as a documented rise in PSA ≥ 2 ng/mL, PSA values > 25% above nadir, PSA elevation in three consecutive determinations at least one week apart, and/or radiological progression in castrated patients with serum testosterone levels < 50 ng/dL [9]. The emergence of CRPC is due to the development of resistance to therapy in cancer cells. CRPC may be due to primary resistance to ADT or acquired resistance when on ADT. This process is androgen-dependent, and androgen receptor (AR) plays a vital role in the development of CRPC. Multiple mechanisms have been proposed, including AR amplification and hypersensitivity, AR mutations leading to promiscuity, coactivators/corepressors, androgen-independent AR activation, and intratumoral and alternative androgen production [10]. The cases of mCRPC are treated with taxane-based chemotherapy in addition to second-generation ADT like abiraterone and enzalutamide [9]. Another response to bypass ADT and/or chemotherapy in prostate cancer is the transformation of histopathology from AR-expressing prostate adenocarcinoma to AR-negative small-cell or large-cell neuroendocrine carcinoma histology, commonly referred to as NEPC [11]. The transformation of histopathology is also referred to as neuroendocrine differentiation of prostate cancer. NEPC is an aggressive variant of prostate cancer that develops later as a mechanism of treatment resistance. It is characterized by down-regulation of PSMA expression with low or non-rising PSA levels [12]. Most NEPCs are a treatment-emergent response to therapy and are termed t-NEPC. Rarely NEPC can also occur as a primary form of prostate cancer with an incidence rate of 1% [13]. Like other neuroendocrine tumors, NEPC is characterized by the expression of neuronal markers, including enolase 2, chromogranin A, and synaptophysin [14]. Serum markers like chromogranin A and neuron-specific enolase are typically raised in NEPC. These also express SSTRs; hence, radiolabeled somatostatin analogs are used in imaging these tumors. The index case presented here in this article is a case of t-NEPC presenting as metastatic disease detected on 68Ga - DOTANOC PET/CT. DOTANOC binds to somatostatin receptor subtypes 2, 3, and 5 expressed on neuroendocrine tumor cells and is hence used as a PET radiotracer agent. NEPC is an aggressive type of mCRPC with a poor prognosis and very often presents with visceral metastases. Treatment guidelines for NEPCs are not clearly defined and mainly consist of platinum-based chemotherapy, which is effective for a short duration. Platinum-based palliative chemotherapy regimens with etoposide and taxanes have been used in treatment. Surgery and radiotherapy have also been used for clinically localized disease or palliation of individual metastatic sites [15]. Targeted radioimmunotherapy agents for NEPC are also being developed for human testing and might be the future of the treatment of these aggressive cancers [16]. It is worth mentioning another rare histological variant of prostate cancer, a mixed small neuroendocrine tumor/acinar adenocarcinoma variant that exhibits aggressive behavior. Molecular imaging to assess PRRT eligibility has been used in such cases. However, the uptake of PET radiotracers targeting PSMA and SSTRs may be reduced and can potentially lead to false negative findings in these cases. This variant of prostate cancer is FDG avid and FDG PET should also be used in these cases [17].

## Conclusions

mCRPC has a treatment-evasive response to ADT in cases of adenocarcinoma prostate. Another mechanism of developing resistance to therapy is neuroendocrine differentiation in these tumors with a resultant transformation of histology to neuroendocrine malignancy from that of adenocarcinoma. These treatment-emergent NEPCs are aggressive forms of mCRPC presenting with distant metastasis, associated with poor prognosis and survival rates. This case study highlights the role of 68Ga - DOTANOC PET/CT imaging in detecting and staging NEPC in a case of CRPC.
